# Organoid transplantation in the adult endometrium restores fertility and uncovers epithelial lineage plasticity

**DOI:** 10.64898/2026.08.28.747350

**Published:** 2026-08-31

**Authors:** Dhanvika Mopure, Hong Im Kim, Claire J. Ang, Daniel J. Davis, Thomas E. Spencer, Kara L. McKinley, Andrew M. Kelleher

## Abstract

The adult endometrium regenerates repeatedly, yet the cells and mechanisms that rebuild its epithelium remain poorly defined. To control the cell types available for regeneration, a genetic model to extensively ablate the uterine epithelium was combined with transplantation of lineage-labeled organoids. Ablation without organoid transplantation triggered re-epithelialization, but resulted in infertility. Transplanted endometrial epithelial organoids engrafted into the ablated uterus, reconstructed both the luminal and glandular epithelia, and restored fertility. Depleting organoids of the glandular lineage before transplantation revealed that luminal epithelial-derived cells acquire glandular identity and function after engraftment. The same luminal-to-glandular epithelial differentiation trajectory emerged during endogenous repair following targeted glandular ablation. Together, these findings establish luminal-to-glandular epithelial conversion as an intrinsic regenerative property of the adult uterine epithelium and establish an endometrial organoid transplantation platform with therapeutic potential.

## Full Text Availability

The license terms selected by the author(s) for this preprint version do not permit archiving in PMC. The full text is available from the preprint server.

